# Genetic counseling for hereditary cancer syndromes: a 5-year experience from a single center in Bulgaria

**DOI:** 10.3389/or.2025.1605606

**Published:** 2025-07-25

**Authors:** Mari Hachmeriyan, Mariya Levkova, Dinnar Yahya, Milena Stoyanova, Eleonora Dimitrova

**Affiliations:** ^1^ Medical University Varna, Department of Medical Genetics, Varna, Bulgaria; ^2^ Laboratory of Medical Genetics, University Hospital “Sveta Marina”, Varna, Bulgaria; ^3^ Medical University Varna, Department of Oncology, Varna, Bulgaria

**Keywords:** hereditary cancer, tumor predisposition syndromes, genetic counseling, genetic testing, HBOC, Lynch Syndrome

## Abstract

This study presents a 5-year retrospective analysis of genetic counseling (GC) services for hereditary cancer syndromes (HCS) at a single center in Bulgaria. The aim is to describe the demographic and epidemiological characteristics of patients seeking GC, the uptake of genetic testing, and the spectrum of identified pathogenic variants. The results highlight an increasing trend in GC utilization. Key findings include differences in patient profiles between those seeking general HCS assessment and those undergoing tumor biomarker testing, the impact of financial accessibility on genetic testing uptake, and a pathogenic variant detection rate of 28% in tested individuals. The most frequently identified conditions were Hereditary Breast and Ovarian Cancer Syndrome and Lynch Syndrome, with pathogenic variants detected in genes such as *BRCA1, MSH2, PALB2,* and *STK11*. These findings underscore the need for enhanced awareness, improved financial access to testing, and the establishment of systematic cascade screening programs in Bulgaria.

## 1 Introduction

Hereditary cancer syndromes (HCS) are characterized by an increased risk of developing certain cancers due to inherited genetic mutations. Genetic counseling (GC) plays a crucial role in identifying individuals at risk, providing risk assessment, discussing genetic testing options, and facilitating informed decision-making regarding prevention and management strategies. The process involves multiple stages, including counseling that occurs prior to a decision on testing, known as pre-analytical GC, which is essential for obtaining informed consent. Understanding the characteristics of patients seeking GC and the outcomes of genetic testing in specific populations is essential for optimizing service delivery and improving patient care. This study aims to describe the 5-year experience of a single center in Bulgaria in providing GC for HCS, focusing on the demographic and epidemiological features of the patients undergoing GC and subsequent genetic testing.

## 2 Materials and methods

This was a retrospective observational study conducted at a single medical genetics center in Bulgaria. Data were collected from the records of patients who underwent genetic counseling between 2020 and 2024. The study included two main groups of patients: those who received GC for the evaluation of potential underlying hereditary tumor predisposition syndromes (HTPS) (n = 154) and those who underwent molecular genetic testing of tumor tissue with subsequent counseling for potential germline implications (n = 157). Data collected included patient demographics (age, gender, geographical location), referral source, risk factors (personal and family history of cancer), type of cancer, the scope of genetic tests performed (e.g., targeted panels, exome sequencing), and the identified genetic variants. Data analysis was descriptive, summarizing the characteristics of the study population and the outcomes of GC and genetic testing using frequencies and percentages.

## 3 Results

To provide a clearer overview of the patient populations, their demographic and clinical characteristics are summarized in Table 1.

**TABLE 1:** Demographic and clinical characteristics of patient groups.

Characteristic	Group 1: Direct GC for HTPS (n = 154)	Group 2: GC post-biomarker testing (n = 157)	Subgroup: Underwent DNA analysis (n = 76)
Mean Age (years)	50	58	47
Gender	66% Female	39% Female	85% Female
Primary Indication	67% Personal Hx of Cancer/Polyposis	100% Personal Hx of Cancer	52% Personal Hx of Cancer/Polyposis
	18% Family Hx only	57% had additional Family Hx	31% Family Hx only
	5% Proactive/No indications	-	10% Proactive/No indications
Primary Cancer Type	Breast, Ovarian, GI/Polyposis	Colorectal, Lung	Breast, Ovarian, GI/Polyposis
DNA Testing Uptake	49% (75/154)	**<1% (1/157)**	100%

### 3.1 Characteristics of patients undergoing GC for potential HTPS

#### 3.1.1 Total number of patients

The number of patients undergoing GC for potential HTPS showed an overall increasing trend from 2020 to 2024, despite a doubling in the cost of services after October 2022 and a slight decrease observed in 2024. Approximately 80% of these patients were residents of Varna, with the rest residing within a 130-kilometer radius of the city. The proportion of GC sessions focused on oncogenetics ranged from 4.3% to 7.5% of all GC sessions per year.

#### 3.1.2 Age and gender characteristics

The age distribution of these patients was 56% (n = 86) aged 50 years or younger and 44% (n = 68) aged over 50 years, with a mean age of 50 years. The patients were predominantly females (66%, n = 102) compared to males (34%, n = 52).

#### 3.1.3 Characteristics by risk factor and referral source

The dominant risk factor was a personal history of cancer or multiple polyposis (67%, n = 103). An additional 12 (8%) had a history of more than one malignancy. Healthy individuals seeking consultation due to a positive family history constituted 18% (n = 28) of the group, while only 5% (n = 8), including three individuals from the same family, proactively sought testing without specific indications due to personal interest in preventative health. The majority of patients (80%, n = 123) were directly referred by a diagnostic hospital unit, an outpatient physician, or an oncology committee. Self-referrals accounted for 20% (n = 31). There was an observed increase in patients seeking outpatient genetic consultation via out-of-pocket payment (n = 100), while the number of patients referred during hospitalization remained relatively stable (n = 54).

### 3.2 Characteristics of patients undergoing GC following tumor biomarker testing

#### 3.2.1 Total number of patients

The total number of patients undergoing targeted biomarker testing in tumor tissue increased significantly annually. Among these, 26% (n = 157) were additionally consulted for a potential underlying HTPS, with a threefold increase within the last 2 years (from 39 in 2023 to 118 in 2024).

#### 3.2.2 Age and gender characteristics

The age distribution in this group showed 80% aged over 50 years, with a mean age of 58 years. Males predominated (61%) compared to females (39%), which is consistent with the dominant pathologies of colorectal and non-small cell lung carcinoma.

#### 3.2.3 Characteristics by risk factor

Among these patients, the majority (57%, n = 90) had a positive family history in addition to their personal cancer diagnosis. Younger age at diagnosis (<50 years) was reported in 35% (n = 54), and 8% (n = 13) reported more than one malignancy.

### 3.3 Characteristics of patients undergoing DNA analysis

#### 3.3.1 Total number of patients

Of all 311 patients who underwent GC, 24% (n = 76) proceeded with DNA analysis, with only one patient from the second analyzed group. Eighty-three percent of those who underwent DNA analysis were residents of Varna.

#### 3.3.2 Age and gender characteristics

Statistically significantly more females (85%, n = 65) than males underwent DNA analysis, with a mean age of 47 years compared to 53 years for those who did not. A statistically significantly higher number of self-funded consultations (referred to as paid consultations, meaning they were paid out-of-pocket by the patient) (93%, n = 71) were followed by genetic analysis compared to inpatient consultations covered by the hospital (7%, n = 5 out of 54 patients).

#### 3.3.3 Characteristics of performed analyses and detected pathology

Regarding the scope of testing, only 18% (n = 14) of patients chose a narrowly targeted gene panel based on their clinical presentation, while 78% (n = 59) preferred the broadest possible multi-gene panel offered within a similar price range. Three patients (4%) underwent whole-exome sequencing (WES).

A pathogenic/likely pathogenic (P/LP) variant associated with the clinical picture was identified in 28% (n = 21) of the 76 tested patients. The diagnostic yield varied by clinical indication: it was 32% for patients with breast and/or ovarian cancer, 28% for patients with gastrointestinal cancer and/or polyposis, 25% for patients with other cancers, and 25% for clinically healthy individuals.

The spectrum of identified pathogenic variants and their associated syndromes is detailed in Table 2. The most common findings were related to Hereditary Breast and Ovarian Cancer (HBOC) and Lynch Syndrome. A variant of uncertain significance (VUS) was reported in 25% (n = 19) of patients.

**TABLE 2:** Spectrum of pathogenic/likely pathogenic (P/LP) variants identified.

Gene	Reported (P/LP) variant	Associated syndrome	Patient clinical presentation
SMAD4	exon 11-12 partial deletion	Juvenile Polyposis/HHT	Gastric & GI polyposis, liver hemangioma
STK11	deletion ex.1	Peutz-Jeghers Syndrome	Suspected Peutz-Jeghers, polyposis
MSH2	c.1386 + 1G>A	Lynch Syndrome	Synchronous colon adenocarcinomas
APC	c.4564_4565del, p.(Leu1522Lysfs*10)	Familial Adenomatous Polyposis	Multiple colorectal polyposis
MLH1	c.67del p.(Glu23LusfsTer13)	Lynch Syndrome	Colon cancer, positive family history
BRCA1	exon 10, c.3756_3759del (p.Ser1253Argfs*10)	HBOC	Ovarian cancer, positive family history
BRCA1	c.5266dup, p.(Gln1756Profs*74)	HBOC	Breast cancer, strong family history
CHEK2	c.592 + 3A>T	HBOC/HCS	Breast cancer
PALB2	c.2257C>T, p.(Arg753*)	HBOC	Breast cancer
BRCA2	c.682-1G>A	HBOC	Two primary breast cancers
NBN	c.657_661del, p.(Lys219Asnfs*16)	NBN-related HCS (HBOC spectrum)	Breast cancer, positive family history
PMS2	c.904-2A>G, p.(Gly757Arg)	Lynch Syndrome	Ovarian cancer
RET	c.2671T>G (p.Ser891Ala)	Multiple Endocrine Neoplasia 2	Medullary thyroid cancer, family history
NF1	c.5749 + 332A>G	Neurofibromatosis Type 1	Clinical signs of NF1, family history
PTPN11	c.922A>G, p.(Asn308Asp)	Noonan Syndrome	Thyroid cancer, suspected Noonan features
NBN	c.657_661del, p.(Lys219Asnfs*16)	NBN-related HCS (HBOC spectrum)	Healthy + Family History
CHEK2	c.470T>C, p.(Ile157Thr)	HBOC/HCS	Healthy + Family History
CHEK2	c.444 + 1G>A	HBOC/HCS	Proactive testing (Healthy)
CHEK2	c.470T>C, p.(Ile157Thr)	HBOC/HCS	Proactive testing (Healthy)
ATM	c.3886_3889del, p.(pro1296Ilefs*52)	Ataxia-Telangiectasia (HCS)	Healthy + Child with Ataxia
BLM	c.1544dup, p.(Asn515Lysfs*2)	Bloom Syndrome (HCS)	Healthy + Family History

Based on the 21 patients with a diagnosed P/LP variant, 36 first-degree relatives were identified as being at risk. Of these, only 19% (n = 7) underwent GC and subsequent targeted testing as a form of cascade screening. This resulted in the identification of 4 additional healthy carriers of pathogenic variants in the *BRCA1, CHEK2, MSH2,* and *NBN* genes, enabling access to preventative care.

## 4 Discussion

This single-center study in Bulgaria provides valuable insights into the uptake and characteristics of individuals seeking GC for HCS. While the increasing number of patients suggests growing awareness, individuals undergoing oncogenetic counseling represent a small proportion of all counseled patients, with growth primarily in self-funded services. This highlights a critical disparity in access, likely driven by financial barriers.

Our finding that younger women predominate among those seeking GC aligns with previous studies [1]. Conversely, the predominance of older men in the tumor-to-germline testing group highlights an important opportunity to reach an otherwise under-accessed population.

A key finding is the significantly higher rate of genetic testing among individuals who self-funded GC (93%) compared to those receiving it through the hospital system (7%). This stark contrast strongly suggests that cost is a major barrier. This discrepancy underscores the need for policies promoting equitable access.

The 28% pathogenic variant detection rate is comparable to other studies [2]. The identification of variants in clinically actionable genes such as *BRCA1, BRCA2, MSH2, MLH1, APC, and STK11* reinforces the clinical utility of genetic testing in identifying high-risk individuals and guiding personalized management [3–5]. However, the geographical concentration of tested individuals in Varna (83%) underscores the impact of accessibility.

The preference for broader gene panels indicates a patient desire for comprehensive genetic information [6, 7]. The VUS rate (25%), similar to the P/LP detection rate, emphasizes the ongoing need for variant re-analysis, a crucial component of responsible genomic medicine.

The low percentage (5%) of proactive counseling (defined as counseling for healthy individuals without specific high-risk family history, sought for preventative purposes) underscores the need for increased public awareness campaigns [8, 9]. A significant limitation of this study is its retrospective nature, which precluded the collection of data on the clinical impact of genetic testing results, such as the uptake of enhanced surveillance or prophylactic surgeries. Furthermore, the small sample size for certain subgroups limits the generalizability of some findings. These represent important areas for future prospective research.

## 5 Conclusion

This 5-year single-center experience provides valuable insights into the landscape of GC for HCS in Bulgaria. The increasing demand is encouraging, but challenges related to awareness, financial accessibility, and the interpretation of results remain. The diagnostic yield of 28% and the spectrum of identified mutations, particularly in HBOC and Lynch syndrome-associated genes, confirm the urgent need for a national strategy. Future efforts should focus on enhancing public and professional education, improving the affordability of genetic testing, and most critically, implementing a systematic cascade screening program to maximize the preventative benefits of cancer genetic services in the Bulgarian population.

## Data Availability

The raw data supporting the conclusions of this article will be made available by the authors, without undue reservation.
